# Best Farming Practices for the Welfare of Dairy Cows, Heifers and Calves

**DOI:** 10.3390/ani11092645

**Published:** 2021-09-09

**Authors:** Giulia Ventura, Valentina Lorenzi, Francesca Mazza, Gianfilippo Alessio Clemente, Claudia Iacomino, Luigi Bertocchi, Francesca Fusi

**Affiliations:** Italian National Reference Centre for Animal Welfare (CReNBA), Istituto Zooprofilattico Sperimentale della Lombardia e dell’Emilia Romagna “Bruno Ubertini” (IZSLER), Via A. Bianchi 9, 25124 Brescia, Italy; giuliaventura66@gmail.com (G.V.); francesca.mazza@izsler.it (F.M.); g.clemente@izsler.it (G.A.C.); iacomino.claudia@gmail.com (C.I.); luigi.bertocchi@izsler.it (L.B.); francesca.fusi@izsler.it (F.F.)

**Keywords:** best farming practices, positive animal welfare, quality of life, positives and negatives, dairy cows, heifers, calves, management, facilities

## Abstract

**Simple Summary:**

The evolving change in societal attitudes regarding animal care and use has led to two main streams of thought. On one hand, there is the concept of “animal rights”, emphasizing that animals should have the same rights as humans, and as such should never be used to benefit humans (e.g., for food, clothing, entertainment, education, research, and even pet ownership). Animals should be able to live a life free of human interference and exploitation. On the other hand, many people agree that humans are responsible for animals and for their care; animals can be used to benefit humans if properly cared for, and their needs are met; abuse and neglect are banned. This is the “animal welfare” (AW) point of view, based on humane treatment, ensuring the physical and mental fitness of animals, as required by current EU legislation. Now, the point is that to fulfill the basic requirements of animal welfare animals are ensured a life worth living, especially given that they can feel emotions. In view of ensuring not only compliance with minimum legislative requirements, but also optimal farming conditions (above minimum legislative requirements), growing attention is attributed to best farming practices. In this work, a list of best practices proposed by the Italian National Reference Centre for Animal Welfare (CReNBA) has been collected to ensure a good quality of life for dairy cows, heifers and calves kept in intensive rearing systems.

**Abstract:**

The concept of animal welfare (AW) has many meanings. Traditionally, AW has been considered as freedom from disease and suffering. Nowadays, growing attention goes to the concept of “positive animal welfare” (PAW), which can be interpreted within the concept of quality of life (QoL), thinking about a “balance of positives over negatives” and a “life worth living”. In this vision, where the QoL represents a continuum between positives and negatives, the Italian National Reference Centre for Animal Welfare (CReNBA), within the Istituto Zooprofilattico Sperimentale della Lombardia e dell’Emilia Romagna (IZSLER), has developed a welfare assessment protocol for dairy cows, heifers, and calves in loose housing systems, including both animal-based and non-animal-based indicators, in which not only hazards but also benefits are identified. This protocol is part of an integrated monitoring system called “ClassyFarm”, belonging to the Italian Ministry of Health and developed by IZSLER. The aim of this paper is to extrapolate from the mentioned protocol, a list of 38 best farming practices (on managerial and equipment factors) for ensuring a high level of welfare in dairy cattle. All stakeholders (veterinarians, farmers, competent authorities, consumers, etc.) can benefit of these best practices as a guide or toolbox to ensure a life worth living for these animals.

## 1. Introduction

The concept of animal welfare (among humans) has been deeply changing, reaching the awareness that animals can feel emotions and, both positive and negative affective states. In the past, the vision of animal welfare was primarily focused on simply avoiding negative welfare consequences, such as pain, fear, distress, frustration, and diseases. Only a few decades ago, the attention of farmers and veterinarians was only focused on ensuring good health conditions in animals raised for food production. High performance in production and freedom from clinical diseases were considered, especially between zootechnicians and veterinarians, to be indicators for animals in good welfare conditions [1,2]. However, scientific outcomes have made it clearer that animal welfare is something more complex than just health and high productivity; animal welfare actually includes animal health, but freedom from disease does not on its own mean good welfare.

This concept is remarked in the two most widely accepted definitions of animal welfare [3]. (1) “*Welfare is a wide term that embraces both the physical and mental well-being of the animal. Any attempt to evaluate welfare, therefore, must take into account the scientific evidence available concerning the feelings of animals that can be derived from their structure and functions and also from their behaviour*” (Brambell Report (965)). (2) “*Welfare is a state of complete mental and physical health, where the animal is in harmony with its environment*” (Hughes (1976) [4]).

The link between animal health and welfare is also enshrined in Reg. EU 2016/429 (the so-called Animal Health Law): better animal health promotes better animal welfare, and vice versa (recital n°7) [5]. Therefore, simply providing animals with a life free from infectious or non-infectious diseases is no longer acceptable. In fact, when referring to illness and diseases within farming conditions, we know that stress conditions and the mental state of an animal can greatly affect its own health status and immunity responses towards opportunistic bacteria, that nowadays represent most pathogens [6,7,8]. 

For these reasons, the recent Farm to Fork Strategy [9] placed great attention in safeguarding a good level of welfare for farmed animals, to avoid the spread of diseases among them, and consequent use and abuse of antimicrobials.

The concepts of One Health—One Welfare stand behind those statements. “One Health” is a project that promotes close collaboration and communication between different disciplines, thus establishing an integrated (holistic) approach with the aim of preventing and controlling all diseases which may cause epidemics between humans and animals (epidemics and epizootic diseases). This kind of holistic approach aims at maintaining the integrity of our ecosystem, for the benefit of all living beings, and guaranteeing a good level of biodiversity, for environmental survival. This emerging scientific coalition represents the only chance to avoid the current and future issues affecting human beings, the animal kingdom, and the environment, leading to a significant improvement in our lives [10,11].

## 2. The Meaning of Positive Animal Welfare (PAW)

For many years, animal welfare science has primarily focused on negative aspects of animal welfare, seeking the elimination of pain, fear, distress, disease, and other forms of suffering [1]. Following a traditional Western way of thinking, good animal health and welfare (even for human beings) were considered equivalent to a state of absence of disease. However, especially referring to both human and animal welfare, this should be understood as an adaptive process, and not just a state or a stable condition. An animal can experience a good level of welfare to the extent that it is able or enabled to adequately manage, day after day, its own adaptive process to the surrounding environment. When this adaptive mechanism enters a state of suffering, the animal’s welfare level, also in terms of good biological functioning, falls apart [12]. However, because it has become widely accepted that emotions are an integral part of the complex life of animals [13], the concept of Positive Animal Welfare (PAW) has begun to arise from the idea that the “absence of sufferings” and “absence of negatives” are not enough [14]. 

However, PAW should not be considered only the opposite of suffering, since it involves qualitatively different aspects [14]. For instance, the concept of PAW is linked with the idea that animals can experience emotions, both negative and positive [15]. The notion that animals can experience emotions comes from growing evidence derived from scientific areas including ethology, psychology, neuroscience, and animal welfare science. Nowadays, there is a growing interest in how to conceptualize and assess when, where, why, and how animals can feel emotions [16]. However, understanding the nature of positive emotions in animals, in relation to the PAW, seems to be rather arbitrary [14]. Indeed, there is still considerable scientific uncertainty about emotions in animals and, perhaps, particularly positive emotions [15]; for example, we lack a ‘gold standard’ against which to assess and validate a positive or negative emotional state in animals [14]. 

It is crucial to understand how animals communicate their emotional state within their environment, and the use of animal-based indicators to assess the welfare of farm animals is universally accepted [17].

The literature and studies on PAW have enabled a more explicit connection between the capacity for animals to “experience positive emotions” and the implications of this for their welfare [14]. In this sense, despite the classic negative conception, the improvements for animal welfare should be based not only on “what the animals suffer from or lack”, but also on the welfare benefits of providing opportunities for positive experiences [18]. 

Especially worth mentioning, is that in the field of on-farm welfare assessment, traditionally implemented animal-based measures (ABMs) almost always focus on the evaluation of negative welfare adverse effects (e.g., poor body conditions, poor cleanliness, skin lesions, lameness, overgrown claws, etc.). However, considering the ABMs from the Welfare Quality^®^ Project (2009) [19], a group of which were included in the so called “Qualitative Behaviour Assessment” (QBA), which has been suggested to be used for evaluating positive emotional states. QBA relies on the ability of observers to judge and integrate perceived details of animals’ body language and posture into descriptors of low/high arousal and positive/negative valence [20]. Playing activities, affiliative behaviors and some vocalizations appear to be the most promising and convenient indicators for assessing positive experiences in farm animals under commercial conditions [21]. The ability of animals to experience positive emotions in the context of animal welfare does not appear to be the only key concept described in the PAW literature.

Lawrence and colleagues [14] have recently conducted a review on the current literature on PAW. This review has found that PAW literature can be articulated into four different topics:

1. Positive emotions, focusing on the capacity of animals to feel positive emotions (as described above);
2. Positive affective engagement that connects positive emotions and behaviors that animals are motivated to engage in;
3. Happiness that represents the full life perspective of the PAW concept;
4. Quality of life, which gives to PAW an interpretation as an appropriate “balance of positives over negatives” and a “life worth living”.

In the next paragraph, we focus our attention on the last topic; the vision of PAW as Quality of Life (QoL).

## 3. Positive Animal Welfare as Quality of Life: A Continuum from Positives to Negatives

The concept of QoL was introduced for the first time by [18], considering the concept of PAW as a “continuum from positives to negatives” and claiming the idea that a welfare continuum may be considered a valid model for overall welfare assessment. Additionally, the Farm Animal Welfare Council (FAWC) [22] agreed to consider PAW in terms of QoL. Its purpose was to classify the animal’s QoL as three possible scenarios: a life not worth living; a life worth living; a good life. The FAWC report [22] stated that other schemes use four or more levels of classification, while only three has the merit of simplicity and the basic notions that are familiar to a human context. This report also deeply influenced the revision over the completeness of the five freedoms as a welfare framework [23] and led to many scientific studies based on FAWC’s QoL approach, such as the resource tier study [24].

Within the PAW, QoL can be seen as a balance between negative and positive welfare, situated at the higher end of the continuum based on either the animals’ overall emotional state [18] or the available opportunities for the animal to have a good life [22].

There are also other interpretations of the concept of QoL. McMillan and colleagues [25], for example, describes animal QoL as something dominated by emotional experiences where subjective feelings play a preeminent role. In their conception, QoL can be totally defined by emotions and, in this continuum, variance from unpleasant to pleasant experiences, the pleasant experiences overcome the unpleasant ones in case of a good QoL. 

In conclusion, this feature of PAW directly leads to a debate about how to add up different aspects of welfare to a total value [14], and this discussion has emerged in connection with efforts to define comprehensive measures for animal welfare assessment at the farm or flock level [26].

The Italian National Reference Centre for Animal Welfare (CReNBA), within the Istituto Zooprofilattico Sperimentale della Lombardia e dell’ Emilia Romagna (IZSLER), developed several on-farm welfare assessment protocols (for different species and animal populations), where QoL is seen as a continuum between negatives and positives. 

## 4. The Assessment of Welfare and the ClassyFarm Protocol

Currently, pending new updates, there are no specific rules for assessing animal welfare [27] and the Council Directive 98/58/EC [28] for the protection of animals kept for farming purposes is considered general, vague, and non-efficient in protecting animals throughout Europe [29]. It is not based on direct observations of the animals, but rather an evaluation of the environment in which they live and the management practices to which they are subjected. In addition, not so long ago, the assessment of animal welfare was mainly based on the analysis of management and facilities; numerous studies, recommendations, and scientific opinions, with the purposes to avoid impaired animal welfare, focused only on the adequacy of the farm environment, without looking at the animals (e.g., TGI 35 L and TGI 200, created by Bartussek in 1985 [30], proposing an animal needs index: TGI—Tier Gerechtheits Index, translated as ANI—Animal Needs Index).

In this contest, the European Welfare Quality^®^ project was one of the best tools to assess the welfare state of farmed animals [19], since it focused on the integration of animal welfare into the food quality chain and developed protocols to measure, among others, the welfare of dairy cows at a farm level [31].

The Welfare Quality^®^ protocol for dairy cows provided information on most of the main hazards identified in the EFSA Scientific Opinions (but not on those where time limitation prevented collecting them) and stated that animal-based measures (ABMs) are necessary to determine whether or not improvements in welfare intended by the recommendations in the Opinion are achieved [27].

In fact, the animal that has its wellbeing at risk can show precise physical signs that can be apprehended, interpreted, and evaluated to understand its real state of wellbeing. The use of ABMs is directly related to the animal’s experience and its ability to cope with the given environment [32].

ABMs may be measured directly from the animal (e.g., lameness) or indirectly, through the collection of data available on the farm (e.g., annual prevalence of mortality), and for which a correlation with animal welfare has been scientifically demonstrated [27]. Including ABMs in an animal welfare assessment process is critical because, between the inputs from the growing conditions (exposure scenario) and the actual welfare of the animal, it is necessary to consider the ability of animals to deal with their environment [4,27,33].

ABMs could be assumed to be more objective and valid than resource and management-based indicators (also called non-animal-based measures N-ABMs), but they are not as practical and their use could make farm audit schemes complicated, less feasible, and costly. The problems of protocols including mostly direct ABMs is that in some cases they require an in-depth level of training, a long execution time, and do not allow for continuous monitoring of animal welfare conditions [32]. Moreover, in some cases N-ABMs could be more feasible than ABMs and could help identify hazards potentially associated with the observed welfare outcomes [27,34]. For instance, in the case of the welfare criteria “absence of prolonged thirst” in the Welfare Quality^®^ protocol, where the measure “water provision” is for reasons of executability a resource-based measure.

For obtaining a comprehensive assessment of animal welfare, it is fundamental to observe the animals and search for adverse effects in response to their environment, thanks to the collection of ABMs; this is improved if added to the evaluation of hazards from inadequate farm management and facilities. For these reasons, to find a compromise between a truthful tool and a practical one, both ABMs and N-ABMs should be included in a comprehensive assessment protocol for animal welfare; this is what the ClassyFarm method provides.

To develop the ClassyFarm protocol for dairy cows, Bertocchi and colleagues [35,36] carried out a study to determine the gap between research and on-farm applications, in order to define public standards that go beyond the minimum requirements fixed by the current European and Italian legislations, as promoted by EFSA (2012) [27]. The ClassyFarm protocol for dairy cattle concerns different subpopulations (calves, heifers, lactating cows, dry cows, transition cows) and is divided in three different thematic areas:

▪ area A: farm management and staff training (which includes N-ABMs related to animal handling, farm managing, staff training and experience);
▪ area B: housing (in which N-ABMs are used to verify the adequacy of the facilities where the animals are kept);
▪ area C: ABMs (in which the ability of the animals to cope with their environment is assessed using indirect and direct ABMs).

Livestock facilities and equipment, as well as management and environmental hygiene, represent a potential source of risk for animal welfare and for satisfying the physiological and ethological needs of animals. Resource- and management-based measures are more likely to highlight potential risks for reduced welfare in the future and help identify the reasons underlying current animal welfare problems [27]. Indeed, the factors that affect an animal’s welfare include the resources available to the animal (which are assessed with resource-based measures), such as space allocation, housing facilities and bedding material, and the management practices of the farm (which are assessed with management-based measures), such as how often the animals are milked, whether analgesics are used, breeding strategies, etc. [27].

Depending on its characteristics (breed, sex, age, etc.) the animal will respond to these inputs, and the animal’s responses are assessed using ABMs. In risk assessment terminology, these responses (collected through animal-based indicators) are the “consequences” of the exposure to the “risk factors” (both hazards and benefits). A list of all indicators is available at Bertocchi et al. (2018) [35] and on Classyfarm (http://www.classyfarm.it, accessed on 2 July 2021).

The protocol is composed of many observations on a multiple choice checklist. Each question has two or three options for the answer: not acceptable/acceptable or not acceptable/acceptable/excellent.

Each area (A, B, C) contributes with a different impact to the calculation of the final animal welfare score obtained in a given farm [36], and the contribution of each parameter on the area scores was determined thanks to a semi-quantitative hazards and benefits characterization, carried out through expert knowledge elicitation (EKE), as described in Bertocchi et al. (2018) [35]. The total score is expressed on scale from 0% to 100% that comes from the analysis of the measures collected on the farm.

At the end of the entire evaluation process, after inputs are entered into the ClassyFarm system a final document is produced, summarizing the results from the data processed and the critical points eventually founded. The following information are reported:

▪ a list of critical points, or the criteria with non-compliant or insufficient responses;
▪ the overall level of risk, relating to the welfare conditions of the animals kept in a given farm;
▪ the level of risk of the animals in relation to each of the three assessment areas, above mentioned.

The possibility of distinguishing the parameters detected in the areas provided by the ClassyFarm system not only allows for categorization of farms into risk bands, but also appropriately addresses preventive interventions for the main weaknesses of the zootechnical system of each individual company, consequently improving the living conditions of the animals.

The result of the application of this evaluation system identify possible legislative non-conformities, critical points (hazards) not directly associated with minimum legal requirements, benefits for animal welfare, and classify the farms through a global numerical index (0–100%) of wellbeing which (based on the score obtained) expresses the level of risk of the farm:

- high risk: unacceptable/negative/dangerous or stress conditions; this indicates the possibility that some of the animals are experiencing or may run into a negative situation (“distress”), due to the impossibility of fully enjoying one or more of the five freedoms [37];
- controlled/medium risk: acceptable/compliant conditions; this is compatible with the possibility that all animals in the herd can satisfy their five freedoms [37] and do not experience stress conditions;
- low risk: optimal/superior conditions, positive and beneficial, due not only to the animal’s full adaptation to its environment and respect for the five freedoms [37], but also the possibility for the animals to live positive, fulfilling and satisfying experiences, producing “eustress”.

Focusing on the optimal condition level (third level), the ClassyFarm method allows the best practices to be highlighted, promoting optimal well-being conditions. 

In fact, best farming practices are a key element to ensure good animal welfare. A combination of best practices seems to be an effective approach for welfare and delivers a genuine assurance of animal welfare when fully embraced and implemented [38]. The best practices that allow optimal conditions emphasize the capacity for animals to live a good life, which could inspire higher aspirations for animal standards.

## 5. Best Practices within the ClassyFarm System for Dairy Cows, Heifers and Calves

In the following paragraphs, the best practices from the Italian ClassyFarm protocol for dairy cows, heifers, and calves (<6 months of age) in intensive rearing systems are listed. They have been divided taking inspiration by the seven tables proposed by EFSA in 2012 [27] relating to the 105 recommendations considered in EFSA (2009) [39] and EFSA (2012) [27]. Modifications have occurred according to our needs. 

References related to the following best practices are available in Bertocchi et al. (2018) [35].

### 5.1. Section 1 → Best Practices Related to Food and Water

To satisfy animal’s nutritional requirements, animals must be fed in accordance with their body growth, order of parity, physiological state, and level of production. Therefore, a specifically calculated diet is needed for at least each animal group at the farm. 

All animals must have an adequate amount of fiber to ensure normal rumen fermentation; for this reason, concentrates should not exceed 60% of ingested dry matter (DM), when the diet does not contain corn silage, and 50% of the dry matter intake when the diet is made up of at least 15 kg of corn silage (corn silage DM is conventionally considered to be completely made up of forage). 

Facilities for delivering feed and water should be designed, constructed, and installed in such a way as to minimize the possibility of feed and water contamination and negative consequences deriving from competition between animals.

The animal’s diet should be of high-quality regarding ingredients: their origin must be known, and they must be stored in suitable environments to prevent alterations or contamination with toxic or harmful substances [27].

1.1. Diet calculation and feed quality (all animals): a specific diet should be calculated for each group of animals by a nutritionist and revised frequently or at least when raw materials are changed, maintaining a traceability system and proper storage of ingredients.
1.2. Feeding management and frequency of food administration (adult cows and heifers): provide constant access to food during the day, for instance delivering a total mixed ration (TMR) available 24 h/day by using unifeed mixer wagons.
1.3. Feeding management and frequency of food administration in calves (milk and fiber): calves should be fed three or more times a day, every day of the week; using automated calf feeders (properly maintained) could help.
1.4. Space availability at the feed bunk (for all animals): provide a number of feeding places > 120% of the total number of animals (for each specific group), with at least two different access points; otherwise provide summer grazing (for at least 60 days/year).
1.5. Feeding places dimension and accessibility:
  1.5.1. For adult cows: ensure a feeding place dimension ≥68 cm/animal, easy access, and presence of a system to prevent cow suffocation;
  1.5.2. For heifers and calves: ensure a feeding place dimension ≥50 cm/animal, easy access, and presence of a system to prevent cow suffocation.
1.6. Water availability (all animals): water should be given ad libitum to all animals, including calves in individual pens feeding milk; water should be clean and subjected to annual tests for potability or taken directly from the public water supply (without intermediate water storage tanks). All animals should be allowed to satisfy their water requirements by drinking when and as much as they wish (especially during hot weather conditions or when they are ill); provide water troughs or drinkers so that animals do not need to wait too long for drinking, nor compete for water, and allow them to put their mouths into the water.
1.7. Cleanliness of water points (all animals): provide that all drinkers or water troughs have clean water and are kept in optimal cleaning conditions, free from feces and inveterate food residues.
1.8. Space availability at drinkers or water troughs:
  1.8.1. For adult cows: provide more than one functioning water bowl for 10 animals or more than 7 cm of trough per animal and presence of several water access points.
  1.8.2. For heifers: provide more than one functioning water bowl for 15 animals or more than 5 cm of trough per animal and presence of several water access points.
  1.8.3. For calves: provide more than one functioning water bowl for 14 animals or more than 5 cm of trough per animal and presence of several water access points.

### 5.2. Section 2 → Best Practices Related to Housing and Equipment

Housing and equipment within the barn represent threats to animal welfare. For this reason, many works, research projects, recommendations and scientific opinions focus primarily on the adequacy of housing conditions. Regardless of the farming system used, animals should have the ability to interact socially, express their own affiliative behaviors and related positive emotions, manifest exploration and play activities, and maintain a stable social hierarchy.

2.1. Housing of animals older than 6 months: All animals are loose housed and have access to an outdoor area (exercising area) with a total surface of at least 4–5 m^2^/head or to a pasture (grazing) for at least 60 days/year, with adequate shelter from sun and rain.
2.2. In case of animals reared only outside the buildings: provide artificial shelters (not only naturals ones), easily accessible by all animals, suitable protection of them all from adverse climatic conditions, predators, and risks to their health; minimum dimensions of the available area should be adequate to allow for comfortable lying down of all the animals and their subpopulations.
2.3. Space availability in the lying area inside the barn (no. cubicles/animal): For adult cows (lactating and dry cows) and heifers: provide a number of cubicles >110% of the total number of animals (for each specific group);
2.4. Cubicle dimensions and design: provide a comfortable lying area even if cubicles are used; they should be long and wide enough in relation to the size of the cows. To be sure of the lying area suitability, check either the cubicle dimensions (non-animal-based measures) or observe how many cows successfully occupy them (animal-based measures); at least 2 h after feeding, milking or any other great event that could potentially disturb animals, cubicles should be occupied by more than 70% of the cows.
2.5. Space availability in the lying area inside the barn (m²/animal):
  2.5.1 For adult cows (lactating cows, dry cows and cows at calving): all animals are loose housed and can access a lying area of more than 7 m^2^/head on deep litter;
  2.5.2 For heifers: all animals are loose housed and can access a lying area of more than 4 m^2^/head on deep litter;
2.6. Space availability for calves up to 8 weeks of age (in single pens): single pen size greater (at least >10%) than the minimum legislative requirements (Italian Decree n. 126/2011 transposition of Council Directive 2008/119/EC [40]). All calves should be free to move and never tethered, including when feeding.
2.7. Space availability for calves housed in group pens: all animals should have access to a space greater (at least >10%) than the minimum legislative requirements (Italian Decree n. 126/2011, transposition of Council Directive 2008/119/EC [40]), that is: for animals <150 kg live weight (l.w.) >1.5 m^2^/animal; 150–220 kg l.w. >1.7 m^2^/animal; >220 kg l.w. >1.8 m^2^/animal. All calves should be free to move and never tethered, including when feeding.
2.8. Type of bedding materials (all animals): provide deep litter or cubicles filled with excellent organic material (e.g., straw-bed abundant, non-abrasive, well preserved, absorbent);
2.9. Type of floors in walking areas (all animals): all animals should be free to move on completely intact flooring, well-preserved and with optimal grip, with either soft rubber flooring or concrete floors;
2.10. Facilities for sick animals (all animals): ensure specific facilities for sick animals, if necessary isolated from the rest of the herd (e.g., infectious diseases), provided with well managed deep litter and registration of sick animals; make sure that lactating cows can be milked by a mobile milking unit or can easily have access the milking parlor if it is very close to the sick pen;
2.11. Temperature, humidity and ventilation conditions (all animals): housing and ventilation should be able to provide sufficient air movement for preventing heat stress in summer conditions; in particular, provide adequate temperature and humidity (temperature and humidity index, THI ≤75) by a cooling plant with a THI automatic control system, especially for lactating cows, dry cows and cows at calving; otherwise provide summer pasture (at least for 60 days/year) equipped with shelters; in case of calves, provide good facilities and procedures for keeping animals constantly protected from changes in temperature, humidity and air quality (both in winter and in summer);
2.12. Air quality and gas (NH_3_, CO_2_) concentrations: provide optimal air quality, ensure gas concentration limits of NH_3_ < 10 ppm; CO_2_ < 3000 ppm.

### 5.3. Section 3 → Best Practices Related to Management, including Management at Calving

Farm management is fundamental in ensuring animal welfare and includes all operations involving stockpersons. Although housing and facilities of a farm may, at first glance, seem more important in terms of their effects on animal welfare, dairy cow welfare is influenced to a greater extent by the daily routine activities performed by the staff. Managerial practices, both those that involve direct or indirect interactions with the animals, can promote best welfare consequences even in facilities that appear to be inadequate or outdated.

3.1. Number of stockpersons involved: ensure that daily activities are performed by at least one stockperson (without milking duties) for less than 200 total animals; otherwise at least one stockperson (with milking duties) for less than 80 total animals (with approximately 40 lactating cows).
3.2. Experience and training of stockpersons: ensure that stockpersons have both practical experience and attend training, for example: at least 10 years-experience and pertinent educational qualification (e.g., university diploma or degree in agricultural sciences, veterinary medicine or similar Bachelor’s degrees) or attendance at a recognized training course on dairy cow farming and welfare during the past 3 years.
3.3. Animal grouping strategy: in order to let stockpersons properly satisfy the specific health and welfare needs of each subpopulation of animals within the herd, ensure that animals are divided based on similar age, size (especially for younger animals), production period or other specific needs (especially for lactating and dry cows); for example, adult cows should be divided into additional groups for transition cows, primiparous cows, multiparous cows, early and final stages of lactation.
3.4. Daily inspections of all animals: provide more than two inspections/day, with additional written or computerized recording of events/observations related to animals.
3.5. Cleanliness of floor in walking areas (all the animals): ensure that all walking areas are correctly managed and the floors are clean and dry.
3.6. Cleanliness of bedding material and related management (all animals and especially cows at calving): ensure that the bedding material (deep litter or cubicles) is clean, well-managed, topped up daily and renewed regularly in all the pens.
3.7. Calving area management: ensure that all cows at calving are kept in single pens (at least 10 m^2^) or group pens (with at least >7 m^2^/animal), on clean, dry and well-managed deep litter; in case of single pens, cows should be moved into them just 12–24 h before calving, in case of group pens, cows should be moved into them at least 15 days before the expected date of calving.
3.8. Colostrum management for newborn calves: feeding colostrum as soon as possible after birth (within 1–2 h) or at maximum within the first 6 h of life; feeding an adequate volume (4 L) of high-quality colostrum with a high immunoglobulin concentration (>50 g/L of IgG or >60 g/L of gamma-globulins); control bacterial contamination of colostrum by proper udder preparation, collecting it in a clean container and storing it properly in a refrigerator or freezer. In case of insufficient health status of the mother or insufficient colostrum production, provide a colostrum bank from the healthy cows on the farm (or if necessary, after sanitation treatments) or from colostrum replacement products; the method of feeding the colostrum and maintaining the colostrum bank should be recorded and documented in a good practice manual.
3.9. Treatment of sick or injured animals: given that prompt observation, farmer first aid, veterinary treatment and, when needed, isolation of sick or injured animals are essential, best practice should include providing all stockpersons with related written procedures for proper management for the caring of these animals (e.g., a management plan for cases of mastitis, lameness, downer cows, dystocia, etc.).
3.10. Culling: in case of sick or injured animals with poor prognosis, culling should be a humane solution and must be carried out only by properly trained operators (e.g., veterinarians, persons holding a certificate of competence for slaughter operations, farm personnel or owners with the appropriate level of competence to do so) using adequate, regularly maintained equipment. Best practice would provide all stockpersons with related written procedures for proper management of the killing and related operations, ensuring that animals are protected from any avoidable pain, distress, or suffering.

### 5.4. Section 4 → Best Practices Related to Milking and Udder Health

Udder infections and mastitis are recognized as one of the most critical pathologies in dairy cows, as they have a great impact on animal welfare due to the pain and discomfort they cause, and since they have negative effects on milk production and composition. For these reasons, it is crucial to identify best practices in order to prevent raising infections, starting from the assurance of a good level of cleanliness of the udder, milking machine, and the surrounding environment, regular supervising of the milking routine equipment, and microbiological monitoring of mastitis pathogens in each affected animal.

4.1. Prevention and control of udder infections: to prevent and control udder infections and mastitis is crucial, at least on bulk tank milk samples, performing regular (on a yearly basis) lab analysis for udder pathogens presence. Best practice includes testing individual animals to implement specific programs for control and eradication of pathogens.
4.2. Waiting room and milking parlor design: assure a big waiting room (>1.8 m^2^/cow), with a waiting time <60 min and easy access to the milking parlor or to provide automatic (voluntary) milking systems, through milking robots.
4.3. Milking machine maintenance: ensure that milking machine or milking robots are regularly inspected and maintained by a specialized servicing programs, with scheduled replacement of those parts subject to any kind of wear; all these operations should be demonstrated by written or computerized records.
4.4. Hygiene of milking parlor (or milking robot) and related equipment: ensure that the milking parlor and milking clusters are cleaned and disinfected after each milking session, and guarantee an optimal level of general hygiene in the surrounding environment.
4.5. Milking routine and udder hygiene: staff should be trained for correct use and management of the milking machine, ensuring adequate udder cleansing and hygiene, use of pre- and post-dipping by means of spray or clean dip cups, correct lag time after udder prestimulation.

### 5.5. Section 5 → Best Practices Related to Foot Care Management

Cows are at a high risk of suffering from lameness and foot diseases, which are considered the most important welfare problems as they are extremely painful and cause a huge negative impact on the health and welfare of cows. 

These negative welfare consequences are strictly linked to many factors (management and equipment related) and poor coping of animals with their environment, such as improper facilities, poor hygiene conditions without manure removal, inadequate flooring (slippery or abrasive), poor foot care, failure to use foot baths, unbalanced diet, selection and genetics. 

Best practices should prevent foot disorders and, in addition to guaranteeing proper management and housing conditions, include the disinfecting and trimming of the hooves.

5.1. Locomotion scoring and foot inspection: provide a written prevention program for lameness and, in order to catch lame cows promptly, regularly observe the way they move and walk within the barn; an objective locomotion scoring system is better. Lame adult cows, within the herd, should never be more than 4%.
5.2. Functional hoof trimming: all cows should have functional hoof trimming at least twice a year and additional therapeutic ones if needed; only properly trained personnel should be involved;
5.3. Foot bathing: provide attention to foot hygiene on a weekly basis by using regularly, on all animals, foot sprays or foot bathing for preventive disinfection (e.g., copper sulphate, zinc sulphate, peracetic acid, glutaraldehyde, iodinated and chlorinated disinfectants, etc.).

## 6. The ClassyFarm IT Platform

The Directorate-General of animal health and veterinary drugs (DGSAF) of the Italian Ministry of Health has financed several multidisciplinary projects with the aim of identifying useful indicators for the categorization of farms based on their level of animal health and welfare, biosecurity, antimicrobial usage and related antimicrobial resistance. 

IZSLER, with contribution from the University of Parma, has thus developed an integrated monitoring system called “ClassyFarm” (http://www.classyfarm.it, accessed on 2 July 2021), carrying the same name as the above-mentioned protocol. The ClassyFarm system, located on the ministerial platform for “Veterinary Information Systems” (https://www.vetinfo.it, accessed on 2 July 2021)), is able to merge different information coming from several sources: (i) self-assessments carried out by veterinary practitioners (regarding animal welfare, biosecurity, health conditions and productive performance, nutrition, etc.); (ii) results of official controls carried out by Veterinary Inspectors on animal welfare and pharmacosurveillance; (iii) laboratory diagnostic results by the Italian “Istituti Zooprofilattici Sperimentali” network (on animal health controls, antimicrobial resistance and susceptibility); (iv) records from a complete digitalization of veterinary medicines usage, from their prescription by a veterinary practitioner to their administration to animals, through the introduction of “electronic veterinary prescription” (https://www.ricettaveterinariaelettronica.it, accessed on 2 July 2021). In the near future, these data will be also integrated by animal-based indicators collected at the slaughterhouse, attempting to predict welfare conditions at the farm level. From the collection and processing of the above-mentioned data, scientifically validated numerical indicators were produced in order to categorize Italian farms, firstly of pigs, cattle and poultry, based on their risk in relation to animal welfare, biosecurity, and antimicrobial usage. The national and local planning of official controls for animal welfare and pharmacosurveillance are performed considering such a categorization, prioritizing inspections in those farms with the worst indicators or unknown conditions (except for electronic veterinary prescription outputs).

The novelty of the ClassyFarm system results in a total 360-degree assessment which attempts to cover all aspects related to animal health and welfare.

## 7. Conclusions

A good quality of animal life and protection of their welfare should be ensured not only by repressive actions, such us by official inspections, but most importantly by providing and disseminating best farming practices. Animals must be reared considering their physiological and ethological needs, and only in this way a good level of animal welfare can be guaranteed [41]. Determining good and best practices for farmed animals is certainly a complex exercise, considering that animal welfare science is such a recent discipline, and limited information is available on how animals’ minds work and process past experiences and information.

Currently, it is crucial to focus on the fact that maintaining good animal welfare should be based on many different factors, such as animal living conditions, respect for their needs, and their ability to cope with the surroundings environment [42].

When pursuing risk assessment and risk management applications in animal welfare, it is also essential to have a big database, collecting information from different sources on welfare conditions in the target animal population, to know the current situation and have the chance to make decisions and tackle particular issues.

In this sense, the ClassyFarm system represents a revolution for the veterinary sector, at least, for Italy; but maybe not only there. Indeed, the ClassyFarm system does not only represent a method for risk assessment and categorization of farms, but leads to a guarantee and awareness of compliance with animal welfare criteria in a context where interest in and sensitivity to animal welfare, as well as a rising in ethical sense, are rapidly developing. 

Simultaneously, there is growing interest and attention among consumers to have deep information about the origin of foodstuffs. In this way, voluntary labelling of animal-welfare-friendly products can represent the best way to communicate information on how farmed animals are kept. However, the message reported on these kinds of products should be guaranteed, and the labelling process should therefore be based on a scientific method, shared and approved among all stakeholders.

In this scenario, the ClassyFarm welfare assessment protocol, and especially the list of best practices contained therein, will be the starting point of a new voluntary food labelling initiative in Italy (called Sistema di Qualità Nazionale per il Benessere Animale, National Quality System for Animal Welfare, SQNBA), harmonized at the national level for promoting and certifying animal-welfare-friendly food products [43].

The environmental, economic, and social sustainability of products of animal origin inevitably go through the requalification of farming techniques and the implementation of new and best practices for animal welfare and agricultural sustainability, that farmers can implement.

## Data Availability

All the datasets considered in this study contain sensitive information and cannot be made publicly accessible but they are available with the anonymization of the participants from the corresponding author upon reasonable request. These best practices belongs to the Classyfarm welfare assessment protocol for dairy cows, available on http://www.classyfarm.it (accessed on 2 July 2021).

## References

[1] Fraser D. (2008). Understanding animal welfare. Acta Vet. Scand..

[2] Nicks B., Vandenheede M. (2014). Animal health and welfare: Equivalent or complementary. Sci. Tech. Rev. Off. Int. des Epizoot..

[3] Brambell F.W.R. (1965). Technical Committee to Enquire into the Welfare of Animals kept under Intensive Livestock Husbandry Systems. Report of the Technical Committee to Enquire into the Welfare of Animals Kept under Intensive Livestock Husbandry Systems.

[4] Hughes B.O. Behaviour as an index of welfare. Proceedings of the Fifth European Poultry Conference.

[5] European Parliament, Council of the European Union (2016). Regulation (EU) 2016/429 of the European parliament and of the council of 9 March 2016 on transmissible animal diseases and amending and repealing certain acts in the area of animal health (‘Animal Health Law’). Off. J. Eur. Union.

[6] Dorian B., Garfinkel P. (1987). Stress, immunity and illness—A review. Psychol. Med..

[7] Biondi M., Zannino L.-G. (1997). Psychological Stress, Neuroimmunomodulation, and Susceptibility to Infectious Diseases in Animals and Man: A Review. Psychother. Psychosom..

[8] Asres A., Amha N. (2014). Effect of stress on animal health: A review. J. Biol. Agric. Healthc..

[9] European Commission (2020). Farm to Fork strategy: For a fair, healthy and environmentally-friendly food system. DG SANTE/Unit ‘Food Information and Composition, Food Waste’.

[10] Kaplan B., Kahn L.H., Monath T.P. (2009). The brewing storm. Vet. Ital..

[11] Monath T.P., Kahn L.H., Kaplan B. (2010). One health perspective. ILAR J..

[12] Lazzari D., La Bella M. Psiconeuroendocrinoimmunologia, stress e cure integrate. Proceedings of the Webinar Presentation, La Risposta Immunitaria Innata a Stressori Infettivi e Non Infettivi, Istituto Zooprofilattico Sperimentale del Piemonte, Liguria e Valle d’Aosta.

[13] Proctor H.S., Carder G. (2015). Measuring positive emotions in cows: Do visible eye whites tell us anything?. Physiol. Behav..

[14] Lawrence A.B., Vigors B., Sandøe P. (2019). What Is so Positive about Positive Animal Welfare?—A Critical Review of the Literature. Animals.

[15] Phillips C. (2008). Animal welfare: A construct of positive and negative affect?. Vet. J..

[16] Mendl M., Burman O.H.P., Paul E.S. (2010). An integrative and functional framework for the study of animal emotion and mood. Proc. R. Soc. B Boil. Sci..

[17] Descovich K.A., Wathan J., Leach M.C., Buchanan-Smith H.M., Flecknell P., Framingham D., Vick S.J. (2017). Facial expression: An under-utilised tool for the assessment of welfare in mammals. Altex.

[18] Yeates J., Main D. (2008). Assessment of positive welfare: A review. Vet. J..

[19] Welfare Quality (2009). Assessment Protocol for Cattle.

[20] Wemelsfelder F., Knierim U., Westerath H.S., Lentfer T., Staack M., Sandilands V. (2009). Qualitative behaviour assessment. Assessment of Animal Welfare Measures for Layers and Broilers.

[21] Boissy A., Manteuffel G., Jensen M.B., Moe R.O., Spruijt B., Keeling L., Winckler C., Forkman B., Dimitrov I., Langbein J. (2007). Assessment of positive emotions in animals to improve their welfare. Physiol. Behav..

[22] FAWC (2009). Farm Animal Welfare in Great Britain: Past, Present and Future.

[23] McCulloch S.P., Wathes C.M., Corr S.A., May S.A., McCulloch S.P., Whiting M.C. (2013). Agriculture, Animal Welfare and Climate Change. Veterinary and Animal Ethics: Proceedings of the First International Conference on Veterinary and Animal Ethics, September 2011.

[24] Edgar J.L., Mullan S.M., Pritchard J.C., McFarlane U.J., Main D.C. (2013). Towards a ‘good life’for farm animals: Development of a resource tier framework to achieve positive welfare for laying hens. Animals.

[25] McMillan F.D. (2000). Quality of life in animals. J. Am. Vet. Med Assoc..

[26] Seligman M.E.P., Steen T.A., Park N., Peterson C. (2005). Positive Psychology Progress: Empirical Validation of Interventions. Am. Psychol..

[27] EFSA Panel on Animal Health and Welfare (AHAW) (2012). Scientific Opinion on the use of animal-based measures to assess welfare of dairy cows. EFSA J..

[28] European Union Council (1998). Concerning the protection of animals kept for farming purposes. Council Directive 98/58/EC. Off. J..

[29] Broom D.M. (2017). Animal Welfare in the European Union.

[30] Bartussek H. (1985). Vorschlag für eine Verordnung der Steiermärkischen Landesregierung für den Bereich der Intensivtierhaltung. Osterr. Freiberufstierarzt.

[31] Keeling L. (2009). An Overview of the Development of the Welfare Quality^®^ Assessment Systems. Welfare Quality^®^ Reports No. 12.

[32] De Vries M., Bokkers E., van Schaik G., Engel B., Dijkstra T., De Boer I. (2014). Exploring the value of routinely collected herd data for estimating dairy cattle welfare. J. Dairy Sci..

[33] Broom D. (1986). Indicators of poor welfare. Br. Vet. J..

[34] Lundmark F., Röcklinsberg H., Wahlberg B., Berg C. (2016). Content and structure of Swedish animal welfare legislation and private standards for dairy cattle. Acta Agric. Scand. Sect. A Anim. Sci..

[35] Bertocchi L., Fusi F., Angelucci A., Bolzoni L., Pongolini S., Strano R.M., Ginestreti J., Riuzzi G., Moroni P., Lorenzi V. (2018). Characterization of hazards, welfare promoters and animal-based measures for the welfare assessment of dairy cows: Elicitation of expert opinion. Prev. Vet. Med..

[36] Ginestreti J., Lorenzi V., Fusi F., Ferrara G., Scali F., Alborali G.L., Bolzoni L., Bertocchi L. (2020). Antimicrobial usage, animal welfare and biosecurity in 16 dairy farms in Lombardy. Large Anim. Rev..

[37] FAWC (1979). Five Freedoms. https://webarchive.nationalarchives.gov.uk/ukgwa/20121010012427/http://www.fawc.org.uk/freedoms.htm.

[38] Main D., Mullan S., Atkinson C., Cooper M., Wrathall J., Blokhuis H. (2014). Best practice framework for animal welfare certification schemes. Trends Food Sci. Technol..

[39] EFSA Panel on Animal Health and Welfare (AHAW) (2009). Scientific Opinion on the overall effects of farming systems on dairy cow welfare and disease. EFSA J..

[40] European Union Council (2008). Council Directive 2008/119/EC of 18 December 2008 laying down minimum standards for the protection of calves. Off. J. Eur. Union.

[41] Oliveira A.F.S., Rossi A.O., Silva L.F.R., Lau M.C., Barreto R.E. (2009). Play behaviour in nonhuman animals and the animal welfare issue. J. Ethol..

[42] Bracke M.B., Hopster H. (2006). Assessing the importance of natural behaviour for animal welfare. J. Agric. Environ. Ethics.

[43] Accredia Presentazione del Nuovo Sistema di Qualità Nazionale per Il Benessere Animale. www.accredia.it/2021/02/12/presentazione-del-nuovo-sistema-di-qualita-nazionale-per-il-benessere-animale/.

